# Rats with high aerobic capacity display enhanced transcriptional adaptability and upregulation of bile acid metabolism in response to an acute high‐fat diet

**DOI:** 10.14814/phy2.15405

**Published:** 2022-08-03

**Authors:** Harrison D. Stierwalt, E. Matthew Morris, Adrianna Maurer, Udayan Apte, Kathryn Phillips, Tiangang Li, Grace M. E. Meers, Lauren G. Koch, Steven L. Britton, Greg Graf, R. Scott Rector, Kelly Mercer, Kartik Shankar, John P. Thyfault

**Affiliations:** ^1^ Molecular and Integrative Physiology University of Kansas Medical Center Kansas City Missouri USA; ^2^ Research Service Kansas City VA Medical Center Kansas City Missouri USA; ^3^ Department of Pharmacology, Toxicology, and Therapeutics University of Kansas Medical Center Kansas City Missouri USA; ^4^ Department of Pediatrics Cornell Medicine New York New York USA; ^5^ Department of Physiology University of Oklahoma Health Sciences Center Oklahoma City Oklahoma USA; ^6^ Division of Gastroenterology and Hepatology University of Missouri Columbia Missouri USA; ^7^ Division of Nutrition and Exercise Physiology Columbia Missouri USA; ^8^ Physiology and Pharmacology The University of Toledo Toledo Ohio USA; ^9^ Anesthesiology University of Michigan Ann Arbor Michigan USA; ^10^ Department of Pharmaceutical Sciences Saha Cardiovascular Research Center, University of Kentucky Lexington Kentucky USA; ^11^ Research Service Harry S Truman Memorial VA Hospital Columbia Missouri USA; ^12^ Arkansas Children's Nutrition Center University of Arkansas for Medical Sciences Little Rock Arkansas USA; ^13^ Department of Pediatrics University of Arkansas for Medical Sciences Little Rock Arkansas USA; ^14^ Section of Nutrition, Department of Pediatrics University of Colorado School of Medicine Anschutz Medical Campus Aurora Colorado USA

**Keywords:** aerobic capacity, bile acids, cholesterol, fatty liver

## Abstract

Rats selectively bred for the high intrinsic aerobic capacity runner (HCR) or low aerobic capacity runner (LCR) show pronounced differences in susceptibility for high‐fat/high sucrose (HFHS) diet‐induced hepatic steatosis and insulin resistance, replicating the protective effect of high aerobic capacity in humans. We have previously shown multiple systemic differences in energy and substrate metabolism that impacts steatosis between HCR and LCR rats. This study aimed to investigate hepatic‐specific mechanisms of action via changes in gene transcription. Livers of HCR rats had a greater number of genes that significantly changed in response to 3‐day HFHS compared with LCR rats (171 vs. 75 genes: >1.5‐fold, *p* < 0.05). HCR and LCR rats displayed numerous baseline differences in gene expression while on a low‐fat control diet (CON). A 3‐day HFHS diet resulted in greater expression of genes involved in the conversion of excess acetyl‐CoA to cholesterol and bile acid (BA) synthesis compared with the CON diet in HCR, but not LCR rats. These results were associated with higher fecal BA loss and lower serum BA concentrations in HCR rats. Exercise studies in rats and mice also revealed higher hepatic expression of cholesterol and BA synthesis genes. Overall, these results suggest that high aerobic capacity and exercise are associated with upregulated BA synthesis paired with greater fecal excretion of cholesterol and BA, an effect that may play a role in protection against hepatic steatosis in rodents.

## Graphical abstract

Here we explored hepatic transcriptional adaptations to a high‐fat diet (HFD) in rats bred for high and low aerobic capacity that display large differences in susceptibility to fatty liver. Rats with high aerobic capacity have greater hepatic transcriptional adaptations with an acute HFD along with increases in BA metabolism that do not occur in rats bred for low aerobic capacity, moreover, these differences seem to be driven by epigenetic signatures.

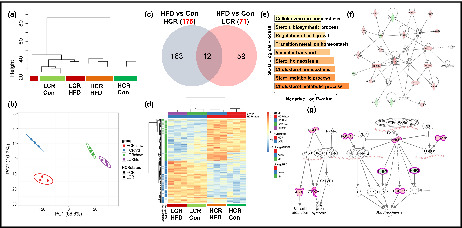

## 1 INTRODUCTION

Nonalcoholic fatty liver disease (NAFLD) encompasses a spectrum of liver pathologies that are increasing at epidemic rates (Younossi et al., 2018). NAFLD is also associated with a greater risk for type 2 diabetes, cardiovascular disease, and advanced liver disease (Marjot et al., 2020). Hepatic steatosis is considered the first step in disease progression, which is marked by excessive lipid storage within hepatocytes (>5%) and is strongly linked to obesity and chronic positive energy balance (Thyfault & Rector, 2020). However, not all obese individuals develop hepatic steatosis suggesting other physiological factors may impact the storage of lipids in the liver (Cusi, 2012; Hodson & Karpe, 2019; Thyfault & Rector, 2020).

Evidence indicates that higher physical activity levels, regular exercise behavior, and/or elevated aerobic capacity reduce the risk for hepatic steatosis, independent of obesity status (Thyfault & Rector, 2020). Previous findings from our lab demonstrate that physical activity via voluntary wheel running (VWR) or treadmill exercise can both prevent and treat hepatic steatosis in rodents (Linden et al., 2015; McCoin et al., 2019; Rector et al., 2008; Rector & Thyfault, 2011). Clinical studies in humans have also shown that exercise training can reduce hepatic lipid storage in as little as 2–4 weeks (Winn et al., 2018).

A widely employed animal model used to evaluate the links between exercise‐ and metabolic‐related disease risks are genetically based rat models selectively bred for low and high intrinsic aerobic exercise capacity (Koch & Britton, 2001). We previously utilized that this contrasting animal model system of low‐capacity (LCR) and high‐capacity runners (HCR) to study the effects of intrinsic aerobic capacity on metabolic outcomes and liver steatosis. As a result of selective breeding, HCR and LCR rats retain dramatic differences in performance on a treadmill exercise running test (more than eight‐fold) in the untrained, sedentary condition (i.e., no access to wheels or treadmills) (Koch & Britton, 2017). Outcome measures in the HCR/LCR rat models have largely mimicked the impact of low versus high aerobic exercise capacity on human health (Thyfault & Morris, 2017; Wisloff et al., 2005). LCR rats fed a normal chow diet show multiple cardiovascular and metabolic syndrome risk factors at a young age (Wisloff et al., 2005), along with early mortality (Koch et al., 2011). Additionally, we have found that LCR rats display hepatic steatosis at a young age on a chow diet, which is further exacerbated by acute (3 days) and chronic (16 weeks) high‐fat/high‐sucrose diet (HFHS) (Morris et al., 2014, 2016). In contrast, HCR rats are resistant to dietary induction of hepatic steatosis, matching the independent effect of aerobic capacity on steatosis risk measured in clinical and epidemiological studies (Church et al., 2006).

The contrasting differences in susceptibility for hepatic steatosis between HCR and LCR rats provide an experimental platform to explore mechanisms impacting the storage of liver fat in hypercaloric conditions. By phenotyping the early metabolic responses to an acute HFHS diet between HCR and LCR rats, we have identified pronounced differences in whole‐body substrate utilization (fat oxidation) and trafficking (lipid and glucose transport to adipose, skeletal muscle, and liver) measured utilizing whole‐body tracer methods, indirect calorimetry, and hyperinsulinemic‐euglycemic clamps (Morris et al., 2014, 2019; Thyfault et al., 2009). We have also reported greater hepatic mitochondrial content, respiratory capacity, and fat oxidation in HCR compared with LCR rats (Morris et al., 2014; Thyfault et al., 2009), matching the previous findings of exercise training studies in mice that demonstrate similar impacts on mitochondrial content, respiration, and fat oxidation postexercise training compared with sedentary controls (Fletcher et al., 2014; McCoin et al., 2019; Rector et al., 2008; Von Schulze et al., 2018). In almost all cases, the HCR rats possessed a greater metabolic rate and/or a greater capacity to enhance specific metabolic traits after the acute onset of an HFHS diet compared with the LCR rats. Britton and Koch et al. have elegantly described the theoretical connection between intrinsic aerobic exercise and enhanced biological capacity which is termed the “Energy Transfer Hypothesis” (Koch & Britton, 2017). Considering this, we hypothesized that in response to acute HFHS, the HCR rats would have a greater capacity to modify hepatic gene transcription in response to acute HFHS compared with LCR, and further, that unique pathways may underlie the ability of HCR rats to respond to the HFHS diet challenge.

Previous results in skeletal muscle between the HCR rats and LCR rats showed pronounced epigenetic regulation of genes (Overmyer et al., 2015). Thus, we hypothesized that baseline hepatic epigenetic variation may also exist between HCR versus LCR rats. Herein, we tested the hypothesis that HCR and LCR rats possess different baseline hepatic gene expression and epigenetic profiles in addition to varied capacities in adaptive gene transcription following an acute HFHS diet. Our findings revealed that the HCR rats display upregulation of hepatic genes controlling the cholesterol and bile acid (BA) synthesis pathway on an HFHS. BA gene expression between strains was also tracked with serum and fecal BA concentrations. HCR/LCR differences in BA metabolism appear to be partially due to differential epigenetic regulation. Furthermore, we show exercise training in male Sprague Dawley rats and female C57BL6J mice also increased hepatic BA genes. Our findings suggest greater transcriptional adaptability of HCR livers to nutrient excess/HFHS compared with LCR livers and implicate a potential role of the BA synthesis pathway in the protection against hepatic steatosis.

## 2 MATERIALS AND METHODS

### 2.1 Animals

The HCR/LCR rat models were developed and characterized as previously described (Koch & Britton, 2001; Noland et al., 2007; Thyfault et al., 2009; Wisloff et al., 2005). At 25–30 weeks of age, male animals were singly housed and acclimated to a low‐fat, control diet (D12110704, 10% kcal fat, 3.0% kcal sucrose; Research Diets, Inc.) for at least 10 days prior to half of the rats being transitioned to ad libitum access of the 3‐day HFHS diet (D12451, 45% kcal fat, 17% kcal sucrose; Research Diets, Inc.). The Institutional Animal Care and Use Committee at the University of Missouri and the University of Kansas Medical Center approved the animal protocols. Liver microarrays were performed in frozen HCR/LCR livers previously described (Morris et al., 2014). Rats were housed in typical vivarium conditions (12/12 h light cycle, room temperature) in a sedentary condition (no access to running wheels) and were provided ad libitum access to food but were then fasted for 12 h in the morning prior to sacrifice. The second cohort of HCR/LCR rats 20–25 weeks of age were used to examine if BA and cholesterol levels were indeed different between HCR/LCR strains. This additional cohort underwent the same ad libitum control and 3‐day HFHS paradigm with the addition of laboratory staff collecting all visible fecal samples each day prior to and during each day of the 3‐day HFHS. On the third day of HFHS, rats were euthanized following a 12‐hour fast using approved methods as described previously (Morris et al., 2014) and remaining feces was collected for analysis. Immediately prior to euthanasia, whole blood was collected first from the portal vein and then from a heart puncture (systemic circulation), centrifuged, and serum was stored at −80°C. Frozen livers from a previous study examining different modes of exercise on hepatic metabolism in male Sprague Dawley rats were also used to obtain mRNA (Fletcher et al., 2014). Last, liver mRNA was also obtained from female C57B/6 mice (Jackson Laboratory) following 4 weeks of VWR from 12 to 16 weeks of age. Rats and mice undergoing wheel running had wheels removed for the last night and thus were euthanized 24 after their last night of VWR. They were also exposed to overnight food restrictions prior to euthanasia. All rodents described were anesthetized with pentobarbital sodium (100 mg/kg) before being euthanized with exsanguination and removal of the heart. In each cohort of rodent studies, treatment groups (VWR vs. Sed or HFHS vs. control diet) were assigned to groups randomly. Primarily male rats were studied as female rats are protected from hepatic steatosis which is a primary outcome of the study.

### 2.2 Hepatic gene expression analysis

A rat (liver) Genome 430 2.0 array was performed by the Genome Technology Access Center at Washington University in St. Louis, MO as described previously (Morris et al., 2016). Briefly, RNA was isolated using TRI reagent followed by clean‐up using Qiagen mini columns including an on‐column DNAse digestion step. Biotinylated cRNA were prepared according to the standard Affymetrix protocol from 100 ng total RNA (Expression Analysis Technical Manual, 2008; Affymetrix). Following fragmentation, 12.5 μg of cRNA were hybridized for 16 h at 45°C on Rat Genome 430 2.0 microarrays. GeneChips were washed and stained using the Affymetrix Fluidics Station 450 and scanned using the Affymetrix GeneArray Scanner 7G06. All analyses were performed using R statistical software (version 4.0) and RStudio (version 1.4). Gene expression differences between groups were determined using the limma package in R following RMA normalization (Ritchie et al., 2015). Pairwise contrasts between HFHS and control diet groups were performed in HCR and LCR rats separately. Transcripts were filtered for *p* < 0.05 and a minimum ±1.5‐fold change in gene expression. Since only four transcripts passed the threshold of significance (*p* < 0.05) following adjustments for multiple‐testing, nominal (raw) *p* values were utilized for filtering. However, both adjusted and unadjusted *p* values are included in the results table. Principal components analysis of normalized data and hierarchical clustering were performed using the Clustviz package (Metsalu & Vilo, 2015). Overlap of transcripts between comparisons was assessed using Jvenn (Bardou et al., 2014). Gene ontology (GO) analysis among differentially expressed genes for biological processes (BP) terms was performed using Enrichr (Kuleshov et al., 2016) and Ingenuity Pathway Analysis (IPA) software (Qiagen) using default parameters as inputs. IPA analysis included identifying top interacting networks based on IPA‐curated knowledge‐base from the known literature. Finally, we carried out gene set enrichment analysis (GSEA) to identify BP, and KEGG pathways enriched by the HFHS diet in each of the groups. GSEA does not rely on an arbitrary cutoff (such as fold change between groups) and is a computational method that determines whether an a priori‐defined set of genes shows statistically significant, concordant differences between two biological states (Subramanian et al., 2005). A ranked gene list method was used to perform testing for the enrichment of *t* statistics in GO and gene sets with a nominal *p* < 0.001 and an false discovery rate (FDR) *q* < 5% being considered as significant per the recommendations of the GSEA software manual.

### 2.3 mRNA expression

RNA and cDNA were prepared as previously described in rat and mouse livers (Morris et al., 2013). Real‐time quantitative PCR analysis was performed utilizing Prism 7000 and SYBR green rat or mouse primers (Table S1; private sharing link for figshare data: https://figshare.com/s/34e64983abf993b36eef). Gene expression was normalized to cyclophilin B and expressed as a fold change compared with the control group.

### 2.4 Hepatic ChIP‐seq

The genomewide localization of histone modifications, spanning varying transcriptional states was analyzed via ChIP‐seq in liver tissue from HCR and LCR rats (only fed state; ChiP‐seq results; publically available DOI for figshare data: https://doi.org/10.6084/m9.figshare.17050337). Liver tissue (100 mg) from 7 to 9 rats/group was pooled, powdered in liquid nitrogen, and cross‐linked with 1% formaldehyde for 10 min. Nuclei were isolated following dounce homogenization in ChIP cell lysis buffer (5 mM PIPES, 85 mM KCl, 0.5% NP‐40). Nuclei were resuspended in ChIP sonication buffer (1× phosphate‐buffered saline, 1% NP‐40, 0.5% sodium deoxycholate, 0.1% sodium dodecyl sulfate [SDS]), and chromatin was sheared using Covaris S220 ultrasonicator (Covaris). Prior to immunoprecipitation (IP), a 50 μl aliquot of sheared chromatin was removed and stored at −70°C to be utilized as an input. Aliquots of sheared chromatin were incubated with antibody‐bound magnetic protein A/G beads (Millipore). Antibodies for H3K4me1 (Abcam; ab176877), H3K4me3 (Abcam; ab8580), H3K9ac (Abcam; ab32129), H3K27ac (Abcam; ab4729), and H3K27me3 (Abcam; ab6002) were incubated with ChIP‐grade protein A/G magnetic beads (2–25 of each antibody ug used per 25 μg of chromatin). All antibodies were ChIP grade. Antibody–chromatin complexes were washed serially in low‐salt, high‐salt, and LiCl‐containing wash buffer and eluted in elution buffer (1% SDS, 0.1 M NaHCO_3_). Immunoprecipitated DNA was purified using standard phenol/chloroform/indole‐3‐acetic acid (IAA) following overnight reverse cross‐linking and treatment with RNaseA and proteinase K (Life Technologies). Immunoprecipitated DNA was quantified using Qubit dsDNA reagents. Purified immunoprecipitated and input DNA were utilized to prepare libraries for sequencing using NEBNext reagents. Briefly, 150 ng of DNA was used for end‐filling, dA‐tailing, and ligation with Illumina's paired‐end adapters using the manufacturer's recommendations. Adapter‐ligated DNA was amplified using PCR and products around 200–350 bp were used for cluster generation. Quantification of the ChIP‐seq libraries was done via the Qubit dsDNA HS Assay kit.

### 2.5 Sequencing data analysis

Single‐read 75‐bp sequencing of libraries was performed using Nextseq 500. Read metrics were visualized with FASTQC and manipulation of fastq files was performed using the FASTX toolkit. Reads were demultiplexed, trimmed of adapter sequences, and filtered for quality. Alignment was carried out using Bowtie2. Identification of peaks was performed using Qeseq (Micsinai et al., 2012) which is optimized to identify peaks for a diverse pattern of ChIP‐seq signals (Micsinai et al., 2012). The peaks identified were imported into Seqmonk (Babraham Bioinformatics) for reading enrichment. Comparisons between LCR and HCR groups were made within Seqmonk. Subsequent data analysis includes annotation of peaks with the closest transcription start site (TSS). Enrichment of GO terms for BP was carried out using Enrichr and *p* values were corrected using the FDR method. To assess chromatin states, we utilized ChromHMM, which employs a hidden Markov Model, to segment the genome into combinatorial patterns of histone marks (Ernst & Kellis, 2012; Ernst & Kellis, 2017). These “chromatin states” correlate with specific spatial and transcriptional profiles (active promoter, poised enhancer, etc.) and allow top‐down assessment of changes in chromatin marks at any given location in the genome. Within ChromHMM, aligned data are binarized for the presence or the absence of an epigenetic mark, which is then used to jointly learn chromatin state models using all epigenetic marks. Chromatin states of specific genes were then compared to assess changes in epigenetic modifications.

### 2.6 Hepatic, serum, and fecal BA and cholesterol concentration

The quantification of total BA was performed enzymatically as previously described (Wang et al., 2015). Briefly, total BAs were extracted from dried, ground liver, and feces (~200 mg) in 83% EtOH 1.6 M NaOH at 70°C for 2 h. During the 2‐h incubation, samples were vortexed briefly every 15 min. Samples were then centrifuged at 1300 *g* for 10 min to remove solids and then quantified enzymatically by measuring 3α‐hydroxy BA as previously described (Talalay, 1960). Total cholesterol was determined via commercially available assay kits (Abcam; ab65359).

### 2.7 Statistical analysis

Animals within strain were randomized by weight into the two dietary groups. The effects of diet, aerobic capacity, and their interaction were evaluated via two‐way analysis of variance with post hoc analysis using the least significant difference to test for any specific pairwise comparisons. In some comparisons, an unpaired *t* test was used to compare differences. Statistical significance was set as *p* < 0.05. Statistical analysis was performed using Prism version 9.0 (GraphPad Software). Data are presented as mean and standard error.

## 3 RESULTS

### 3.1 HCR/LCR anthropometrics

For the first cohort of rats, body weight and composition, hepatic triglyceride content, and liver histology of rats used for liver gene expression analysis were reported previously (Morris et al., 2014). The second cohort of rats presented in the current study was used to specifically measure serum and fecal BA. In this set of rats, we found that the acute HFHS diet induced the same impact on body weight and body composition in HCR/LCR rats as found previously in Table 1. In agreement with our previous findings, the LCR rats displayed a greater body weight and retroperitoneal fat/BW ratio compared with HCR rats. Both HCR and LCR rats on the HFHS had greater induction of weight gain over 3 days compared with rats maintained on the control diet; however, the LCR displayed a three‐fold higher rate of weight gain on the HFHS compared with HCR rats. As such, feed efficiency over the 3‐day HFHS period was 2.5‐fold higher in the LCR than in the HCR. Our previous studies indicated greater food and energy intake in LCR compared with HCR during acute HFHS feeding, which was not observed in the current cohort of animals. The lack of observed differences in food and energy intake here may be, in part, due to daily disturbances in the home cage to collect fecal samples. Collectively, we observed similar differences in weight gain induced by acute HFHS as reported previously (Morris et al., 2014, 2016, 2019).

**TABLE 1:** Anthropometrics

	HCR		LCR		Interaction
	Con	HFHS	Con	HFHS	
Final BW (g)	378.2 ± 11.9	383.6 ± 9.9	482.6 ± 19.3*	474.1 ± 19.8*	NS
Three‐day daily weight gain (g)	2.4 ± 1.2	4.4 ± 1.2	1.3 ± 0.8	12.5 ± 2.0** ^,^ ^††^	*p* < 0.003
Retroperitoneal fat/BW (g)	17.6 ± 1.6	20.6 ± 1.5	25.5 ± 1.1*	24.2 ± 2.1*	NS
Three‐day food intake (g)	61.5 ± 1.5	57.0 ± 2.8	55.8 ± 2.2	58.5 ± 3.7	NS
Three‐day energy intake (cal)	236.9 ± 5.6	269.8 ± 13.1^†^	214.7 ± 8.6	276.6 ± 17.3^†^	NS
Three‐day energy intake (cal/g)	631.7 ± 27.7	699.7 ± 43.9	445.6 ± 10.8*	589.0 ± 34.9* ^,^ ^††^	NS
Feed efficiency (mg/kcal)	9.5 ± 4.9	15.8 ± 4.1	8.7 ± 2.0	43.4 ± 5.7** ^,^ ^††^	*p* < 0.006

*Note*: Final body weight, daily weight gain during the 3‐day HFHS, retroperitoneal fat normalized to BW, food intake during the 3‐day HFHS, absolute and normalized energy intake, and feed efficiency. Data are expressed as mean ± standard error. *n* = 8/condition.

Abbreviations: BW, body weight; CON, control; HCR, high‐capacity runner; HFHS, high‐fat/high‐sucrose diet; LCR, low‐capacity runner; NS, not significant.

* *p* < 0.05, main effect, HCR versus LCR

** *p* < 0.05, HCR versus LCR within diet.

^†^ *p* < 0.05, main effect, CON versus HFHS.

^††^ *p* < 0.05, CON versus HFHS within genotype.

### 3.2 HCR/LCR microarray analysis

**FIGURE 1:**
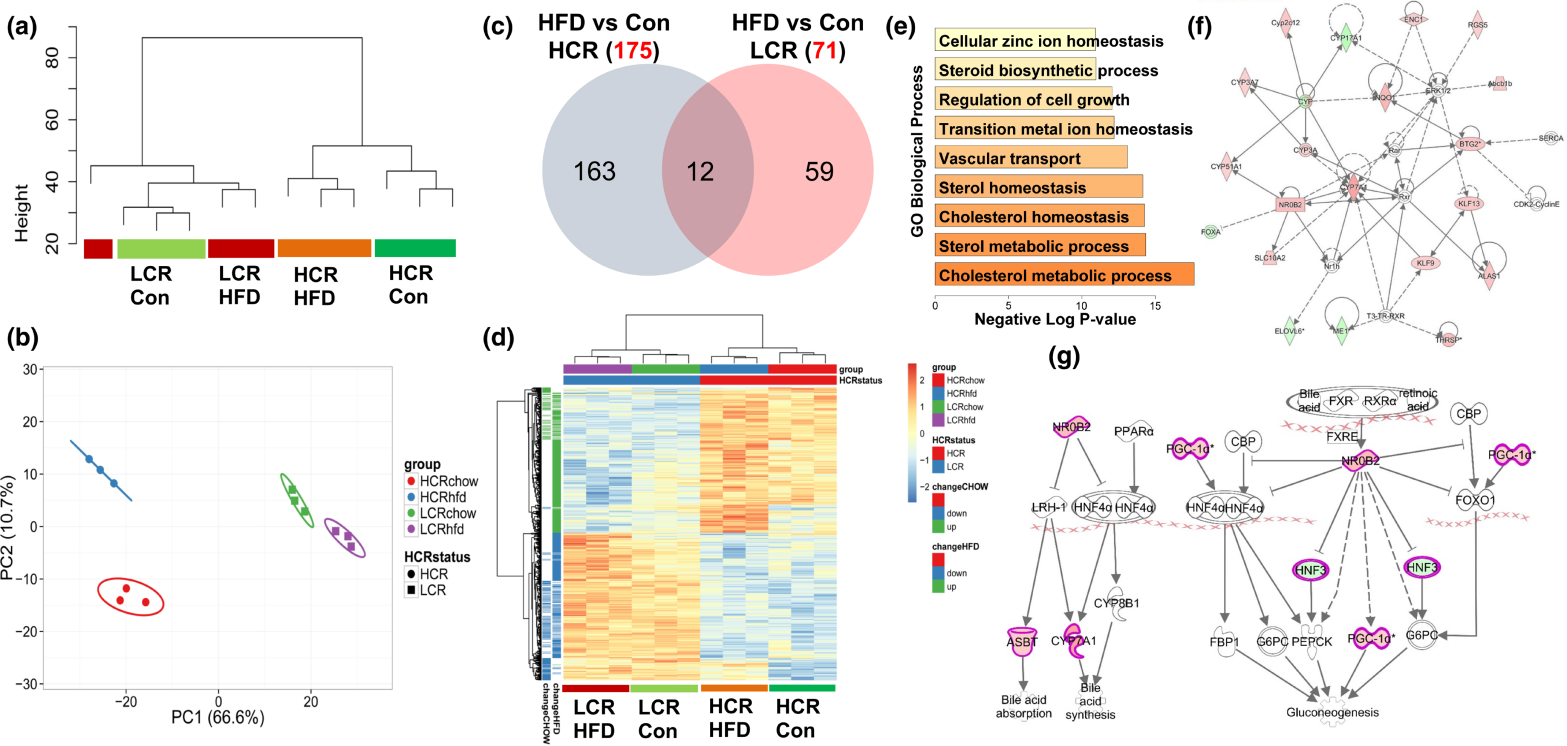
Hepatic microarray, gene ontology, and ingenuity pathway analysis. Gene expression analysis utilizing microarrays of liver tissue from the high‐capacity runner (HCR) and low‐capacity runner (LCR) rats. (a) Global hierarchical condition clustering of samples and (b) principal component analysis of RMA normalized gene expression values. (c) Venn diagram of differentially expressed genes (±1.5‐fold, *p* < 0.05) following 3‐day HFHS in HCR and LCR rats, respectively. (d) Heatmap representing normalized values of the union of differentially expressed genes. (e) Gene ontology (GO) analysis of enriched biological processes among differentially expressed genes in HCR following HFHS diets. (f) Top network of differentially expressed genes involved in sterol and lipid metabolism in HCR following HFHS diets via Ingenuity Pathway Analysis (IPA). (g) Canonical upstream regulators of bile acid and lipid metabolic pathways derived from IPA. *n* = 8/condition. HFHS, high‐fat/high‐sucrose diet.

We performed microarray‐based gene expression analysis of livers from HCR/LCR rats that underwent acute 3‐day HFHS as previously reported (Morris et al., 2014). Unsupervised global condition clustering revealed that expression differences between LCR and HCR rats were greater than acute high‐fat diet effects (Figure 1a). Further, principal component analysis of global gene expression showed both strong effects of genotype (HCR vs. LCR) and a prominent effect of HFHS diet in HCR rats relative to response in LCR rats, suggesting a more robust adaptation to HFHS (Figure 1b). This was reflected in a greater number of significantly altered transcripts on HFHS in HCR animals relative to LCR rats (175 vs. 71, adjusted *p* < 0.05) (Figure 1c) and minimal overlap in genes affected by HFHS diet in both groups (~7% of genes affected in HCR rats). Hierarchical clustering of the union of all differentially expressed genes showed a clear distinction in genes altered in HCR and LCR rats following HFHS diets (Figure 1d). These overall findings suggest that an acute HFHS diet elicits a more robust transcriptional response in HCR compared with LCR rats. We examined the enrichment of GO BP among differentially expressed genes altered in HCR rats in the context of HFHS using Enrichr and IPA software. These analyses clearly showed that genes involved in cholesterol metabolism and homeostasis, sterol and lipid metabolism, and steroid biosynthesis were uniquely affected in HCR animals (Figure 1e). IPA analysis confirmed findings from Enrichr and identified a top network of genes involved in sterol and BA metabolism (Figure 1f). Upstream regulators of key members of the cholesterol/BA synthesis pathway included small heterodimer partner (SHP/NR0B2), Peroxisome proliferator‐activated receptor‐gamma coactivator‐1α (PGC‐1α), and Hepatocyte Nuclear Factor 3/4 alpha (HNF3/4α) genes as identified by IPA analysis (Figure 1g). Finally, analysis via GSEA also revealed unique transcriptional differences between HCR and LCR groups after HFHS diets. KEGG pathways analysis showed that LCR displayed a concerted reduction in expression of TCA cycle genes, results that were not witnessed in the HCR (Figure S1; private sharing link for figshare data: https://figshare.com/s/fcc4568ca28bc0314cce). These included significant reductions in phosphoenolpyruvate carboxykinase (PCK1), aconitase 1 (ACO1), ATP citrate synthase (ACLY), succinate‐CoA ligase 1 and 2 (SUCLG1 and SUCLG2).

### 3.3 HCR/LCR liver gene expression

**FIGURE 2:**
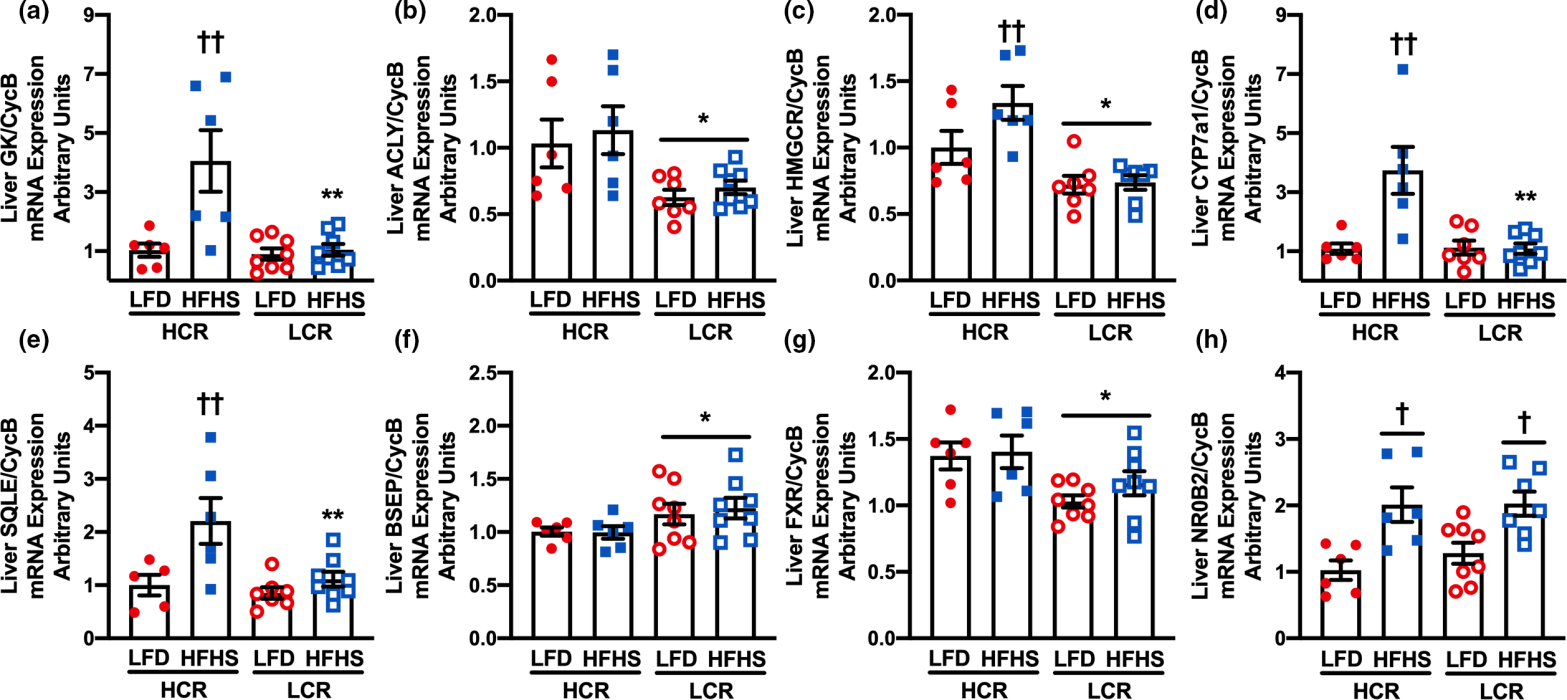
Hepatic gene expression. Hepatic gene expression was measured in high‐capacity (HCR) and low‐capacity (LCR) runners before after an acute 3‐day low‐fat diet control (CON) or high‐fat/high‐sucrose (HFHS) diet intervention. Hepatic gene expression for (a) glucokinase (GK), (b) ATP citrate synthase (ACLY), (c) 3‐hydroxy‐3‐methyl‐glutaryl‐CoA reductase (HMGCR), (d) cholesterol 7 alpha‐hydroxylase (CYP7a1), (e) squalene epoxidase (SQLE), (f) liver bile salt export pump (BSEP), (g) farnesoid x receptor (FXR), and (h) small heterodimer partner (SHP/NRB02). The effects of diet, aerobic capacity, and their interactions were evaluated via two‐way analysis of variance with post hoc analysis using the least significant difference to test for any specific pairwise comparisons. *p < 0.05, main effect, HCR versus LCR; ^†^ *p* < 0.05, main effect, CON versus HFHS; **p < 0.05, HCR versus LCR within diet; ^††^ *p* < 0.05, CON versus HFHS within genotype. Data are presented as mean and standard error. *n* = 8/condition.

We followed up microarray methodologies by measuring gene expression via qPCR for targeted genes that exhibited the greatest differential expression between groups (Figure 2). Liver glucokinase was increased by HFHS in HCR rats only, with no changes observed in LCR rodents (Figure 2a). Gene expression of ACLY was lower in LCR compared with HCR regardless of diet conditions (Figure 2b). Gene expression for both 3‐hydroxy‐3‐methyl‐glutaryl‐CoA reductase (HMGCR, Figure 2c) and cholesterol 7 alpha‐hydroxylase (CYP7a1, Figure 2d) was greater following acute HFHS in the HCR rats only (Figure 2c,d). Squalene exopidase (SQLE) was greater on an HFHS in both groups but achieved statistical significance in the HCR rats (Figure 2e). Gene expression of liver bile salt export pump (BSEP, Figure 2f) was greater in LCR, whereas farnesoid x receptor (FXR, Figure 2g) was lower in LCR during both diet conditions. Last, liver expression of SHP/NR0B2 was uniformly increased by HFHS in both strains (Figure 2h).

### 3.4 Histone modifications in HCR/LCR livers

**FIGURE 3:**
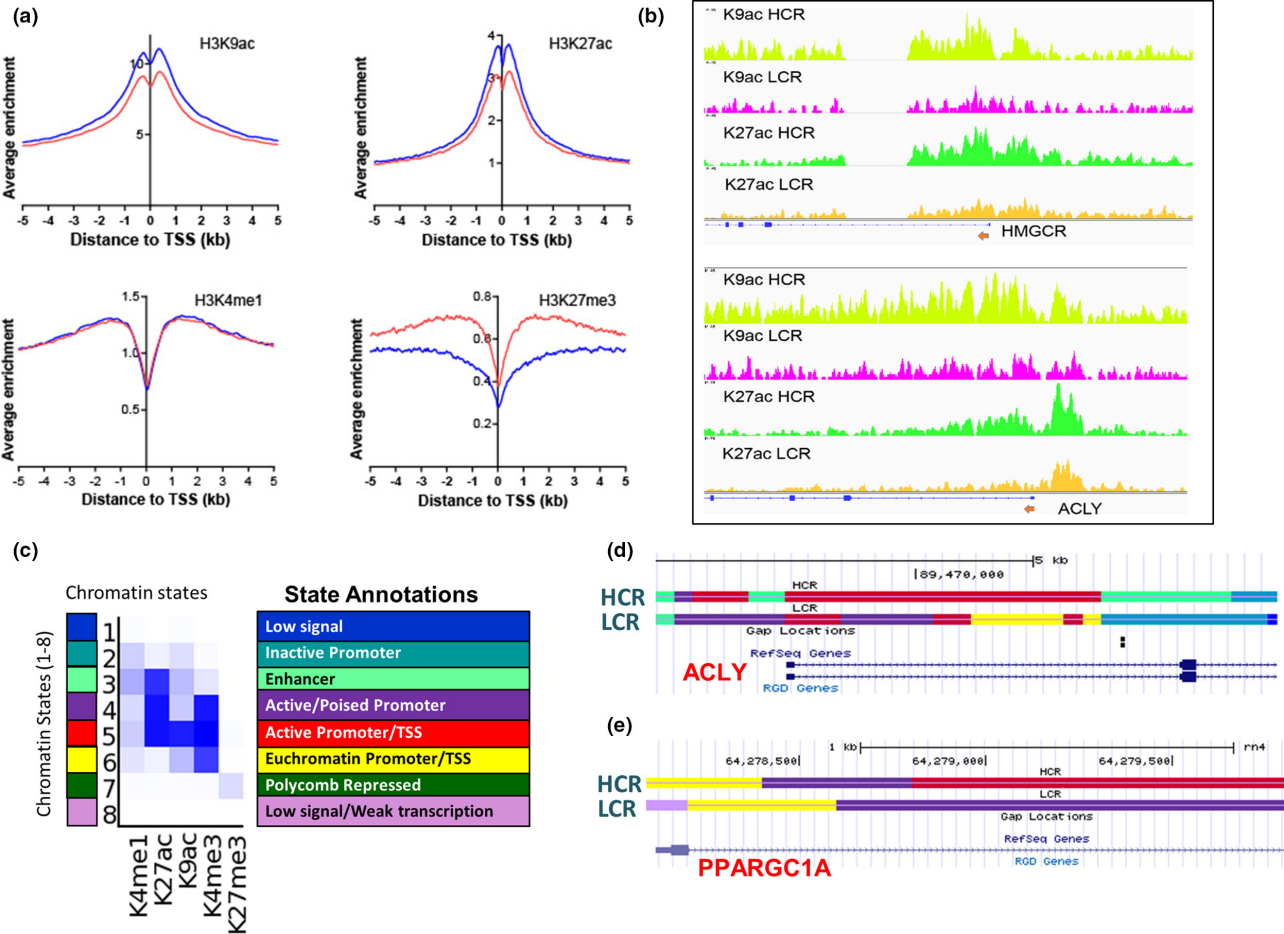
Histone modification in HCR and LCR liver. (a) Average normalized read densities for respective histone marks in ±5 kB regions around TSS in the liver of HCR and LCR. For each group, the average enrichment of reads per base pair (corrected for total counts) represented as log_2_ values, was computed separately. (b) Read enrichment of H3K9ac and H327ac histone modifications around promoters of HMGCR and ACLY genes in HCR and LCR, with HCR showing a greater abundance of histone marks. (c) Genome state assignments following de novo genome segmentation into eight chromatin states with ChromHMM. Chromatin states were learned from ChIP‐seq data using ChromHMM. A heat map represents the enrichment of specific histone marks or combinations in the assigned state (e.g., state 5 is enriched for the cooccurrence of H3K9ac, H327ac, and H3K4me3 marks, typical of active euchromatin regions). (d) Chromatin state comparisons for promoter and genic regions of ACLY and PPARGC1A genes between HCR and LCR groups. Colors of chromatin states (Cusi, 2012; Hodson & Karpe, 2019; Linden et al., 2015; Marjot et al., 2020; McCoin et al., 2019; Rector et al., 2008; Thyfault & Rector, 2020; Younossi et al., 2018) correspond to those in (c) (e.g., red indicates state 5 and yellow indicates state 6). *n* = 7–9 pooled/condition. ACLY, ATP citrate synthase; HCR, high‐capacity runner; HFHS, high‐fat/high‐sucrose diet; HMGCR, 3‐hydroxyl‐3‐methyl‐glutaryl‐CoA reductase; LCR, low‐capacity runner; TSS, transcription start site.

Histone modifications can alter chromatin structure and accessibility which directly impact transcription. We assessed genomewide localization of histone modifications associated with active transcription (H3K4me3, H3K9ac, and H3K27ac), enhancer (H3K4me1), and polycomb‐mediated repression (H3K27me3) in livers from HCR and LCR rats. Annotated genomic locations enriched for each histone modification altered between groups (HCR vs. LCR, min ±2‐fold) are included in Supplementary Data (publically available DOIs for figshare data: https://doi.org/10.6084/m9.figshare.17050343; https://doi.org/10.6084/m9.figshare.17050349). To evaluate broad changes in histone marks, we computed the average read coverage for each histone mark in ±5 kb regions around the TSS for all genes in HCR and LCR groups (Figure 3a). These analyses revealed that HCR livers were associated with a gain in histone acetylation marks H3K9ac and H3K27ac, but no changes in H3K4me1 or H3K27me3 marks. To examine if genes that are transcriptionally altered in HCR animals were also associated with altered histone modifications, we visualized histone mark enrichment on these genes. Promoter regions of HMGCR and ACLY showed greater K27ac and K9ac in HCR animals (Figure 3b). To further confirm these results, we examined chromatin state assignments of these regions from ChromHMM analysis (Figure 3c). In LCR animals, ACLY promoter and genic regions showed active and poised promoter state assignments (States 4 and 5), whereas HCR animals showed strong state 5 throughout the gene (indicative of the presence of H3K4me3, H3K27ac, and H3K9ac marks). Likewise, genic regions of the PGC1α (PPARGC1A) gene also showed State 5 assignment in HCR animals which was absent in LCR animals (Figure 3d,e). Several other genes in the cholesterol metabolism pathway including SQLE and CYP51A1 showed enhanced acetylation in their promoters. Collectively, these findings indicate that the observed changes in cholesterol and BA genes in HCR rats in response to acute HFHS may be facilitated by epigenetic modifications, specifically histone acetylation modifications.

### 3.5 Liver, serum, and fecal BAs

**FIGURE 4:**
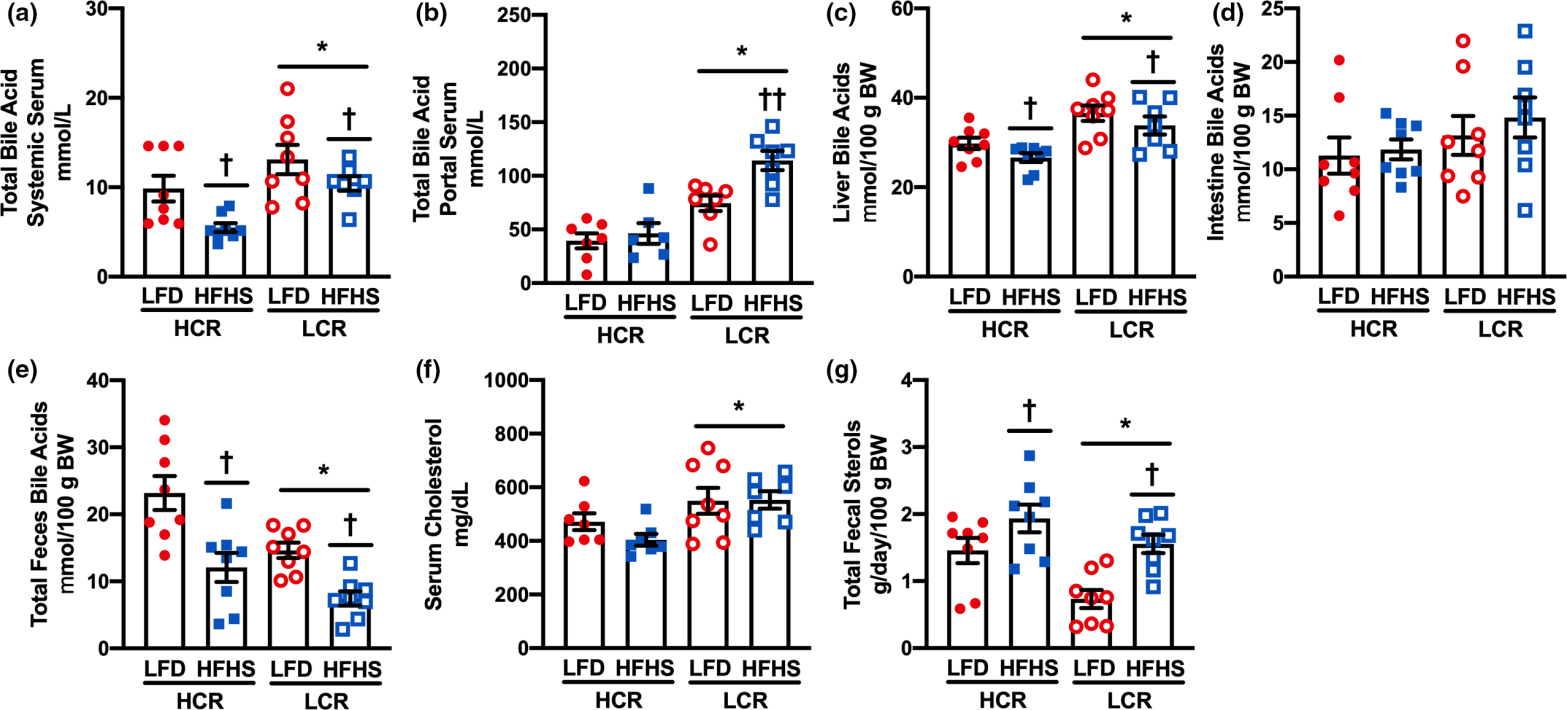
Serum, liver, and fecal bile acids. Bile acid and cholesterol contents were measured in high‐capacity (HCR) and low‐capacity (LCR) runners before after an acute 3‐day low‐fat diet control (CON) or high‐fat/high‐sucrose (HFHS) diet intervention. Bile acid concentrations measured in (a) systemic and (b) hepatic portal serum. Bile acid content was measured in (c) liver (d) intestine, and (e) feces. Cholesterol concentrations were measured in (f) serum and (g) feces. The effects of diet, aerobic capacity, and their interaction were evaluated via two‐way analysis of variance with post hoc analysis using the least significant difference to test for any specific pairwise comparisons. *p < 0.05, main effect, HCR versus LCR; ^†^ *p* < 0.05, main effect, CON versus HFHS; **p < 0.05, HCR versus LCR within diet; ^††^ *p* < 0.05, CON versus HFHS within genotype. Data are presented as mean and standard error. *n* = 8/condition.

We next determined if serum and fecal BA levels were different between HCR and LCR strains to determine if gene expression findings tracked with metabolic differences in BA synthesis and excretion. LCR rats displayed greater BA levels in systemic circulation in both diet conditions, whereas the 3‐day HFHS diet significantly lowered systemic BA levels in both strains (Figure 4a). Portal serum concentrations of BA were also greater in LCR rats compared with HCR, an effect that was further increased by the HFHS diet (Figure 4b). LCR rats exhibited greater liver BA content than HCR (Figure 4c). HFHS reduced liver BA content significantly in both HCR and LCR strains (Figure 4c). Intestinal BA content trended to be higher in the LCR rats over HCR but was not impacted by diet (Figure 4d). HCR rats had greater fecal BA measured over a 3‐day period compared with LCR; however, fecal BA concentrations decreased regardless of strain compared with animals fed a control diet (Figure 4e). Serum cholesterol was greater in LCR rats compared with HCR yet was not altered by acute HFHS in either strain (Figure 4f). Total fecal sterol excretion (combined sterol and BA) was greater in HCR rats and the 3‐day HFHS diet increased total fecal sterol excretion in both groups (Figure 4g).

### 3.6 Liver gene expression following voluntary wheel exercise

The HCR versus LCR model is a proven model to investigate the intrinsic (genetic) effects of aerobic capacity. Aerobic capacity can also be readily modified by exercise training. We, therefore, measured the expression of genes involved in cholesterol and BA synthesis in male rats and female mice following 4 weeks of VWR shown in Table 2. Male rats averaged running 4 km/day, whereas female mice ran on average ~8–9 km/day. Gene expression for ACLY, HMGCR, and CYP7a1 were significantly greater in both male Sprague Dawley rats and female wild‐type C57/BL6J mice following 4 weeks of VWR compared with sedentary controls. Additionally, the greater expression of genes involved in cholesterol and BA synthesis in female mice following VWR was accompanied by a significant two‐fold increase in fecal BA excretion concentration collected over a 7‐day period compared with sedentary control mice (1.0 ± 0.10 vs. 0.5 ± 0.03 nmol/g/BW/day, *p* < 0.05 for VWR vs. sedentary). These collective findings show that exercise also upregulates genes involved in cholesterol and BA synthesis and increases fecal BA excretion.

**TABLE 2:** Liver mRNA expression

	SD rats (M)		C57BL/6J Mice (F)	
	SED	VWR	SED	VWR
ACLY	1.0 ± 0.1	3.1 ± 0.7*	1.5 ± 0.2	1.9 ± 0.1*
HMGCR	1.1 ± 0.1	3.0 ± 0.3*	1.1 ± 0.2	3.0 ± 0.3*
CYP7a1	1.2 ± 0.3	5.6 ± 1.0*	0.9 ± 0.1	1.7 ± 0.1*

*Note*: Liver mRNA expression was measured following 4 weeks of remaining SED or VWR in male (M) Sprague Dawley (SD) rats and female (F) C57BL/6J mice. Data are expressed as mean ± standard error.

Abbreviations: ACLY, ATP citrate synthase; CYP7a1, cholesterol 7 alpha‐hydroxylase; HMGCR, 3‐hydroxyl‐3‐methyl‐glutaryl‐CoA reductase; SED, sedentary; VWR, voluntary wheel running.

* *p* < 0.05 SED versus VWR. *n* = 8–10/condition.

## 4 DISCUSSION

We have previously reported that HCR rats are protected from acute and long‐term effects of nutrient excess on metabolic health (obesity, insulin resistance, and hepatic steatosis), whereas the LCR are highly susceptible. The purpose of this study was to investigate the impact of high (HCR) or low (LCR) intrinsic aerobic capacity on liver‐specific adaptations in response to acute nutrient excess (HFHS) and identify potential mechanisms that impact susceptibility for hepatic steatosis and overall metabolic health. Our results indicate that HCR rats with elevated aerobic capacity display greater hepatic transcriptional adaptations to an acute HFHS diet than LCR rats. In addition, epigenetic signatures controlling transcriptional adaptive capacity are different between the HCR and LCR rats, which may contribute to the differences in transcriptional adaptability. Gene expression differences pointed to a potential role of BA metabolism between strains. Follow‐up qPCR analyses confirmed that HCR rats display upregulation of genes controlling the cholesterol and BA synthesis pathway on an HFHS. The differences in BA gene expression were tracked with metabolic differences in BA between strains. Furthermore, exercise in Sprague Dawley and C57BL6J mice similarly increased hepatic BA genes. Our findings confirm the greater adaptability of HCR livers to nutrient excess compared with LCR and further implicate a potential role of BA synthesis pathways in the protection against hepatic steatosis and overall metabolic health during nutrient excess.

In the postprandial state, hepatic acetyl‐CoA supply is increased via glycolysis and β‐oxidation; this greater supply of acetyl‐CoA results in the export of citrate out of the mitochondria where it is converted back to acetyl‐CoA by ACLY and serves as a substrate for two separate pathways, de novo lipogenesis or cholesterol, and subsequent BA synthesis. BAs are synthesized in the liver and secreted into the small intestine to facilitate lipid absorption and are primarily reabsorbed (~95%) and returned to the liver via the portal vein (Chiang, 2013). Recycled BA feedback to inhibit BA synthesis by activating hepatic FXR (via SHP/NR0B2 signaling) and reducing CYP7a1 expression (Thomas et al., 2008). Chronic states of energetic surplus (i.e., obesity, HFHS diet) and systemic insulin resistance are associated with greater trafficking of acetyl‐1CoA toward de novo lipogenesis, a major contributor to hepatic steatosis via excess lipid storage (~10%–40%) (Donnelly et al., 2005; Smith et al., 2020). Our previous findings align with this by indicating acute HFHS results in robust accumulation of liver triglyceride content and greater hepatic insulin resistance in LCR rats, whereas HCR rats remained protected (Morris et al., 2014). However, it should be noted that hypercaloric methods to induce steatosis in rodents commonly capitalize on high fat in the diet which suppresses de novo lipogenesis but likely still contributes to hepatic lipid storage (Duarte et al., 2014). Our current findings further add to this by demonstrating that HCR rats have greater hepatic transcriptional adaptability and expression of genes involved in cholesterol and BA synthesis (i.e., HMGCR and Cyp7a1, respectively) in response to acute HFHS compared with LCR rodents. The upregulation of genes involved in cholesterol and BA synthesis may suggest greater trafficking of acetyl‐CoA toward BA synthesis rather than de novo lipogenesis. Further work directly measuring nutrient flux to de novo lipogenesis (DNL) and BA synthesis is needed to confirm if this is a critical control point.

There is growing interest in understanding the role of BA metabolism in chronic disease with a specific interest in Cyp7a1, the rate‐limiting enzyme for BA synthesis, as a potential target for therapeutic interventions to prevent hepatic steatosis (Chiang, 2013). For example, genetic overexpression of Cyp7a1 in mice results in a protection against diet‐induced hepatic steatosis paired with greater fecal excretion of BA and altered BA composition (Li et al., 2010). Importantly, Cyp7a1 is regulated by glucose‐ and insulin‐specific mechanisms and becomes dysregulated with diet‐induced obesity and/or insulin resistance (Li et al., 2012). Insulin resistance is also associated with greater systemic concentrations of BA, largely comprised of 12α‐hydroxylated BA (Haeusler et al., 2013). Our current findings align with previous reports by which insulin‐sensitive HCR rats have greater expression of Cyp7a1, lower systemic serum BA concentrations, but higher fecal BA compared with their insulin‐resistant LCR counterparts. Overall, we interpret our findings to suggest a potentially critical role for Cyp7a1 in hepatic energy homeostasis. An interesting caveat is that the 3‐day HFHS lowered fecal BA concentration in both the HCR and LCR strains. The mechanisms underlying this change are unknown, however, we have unpublished data suggesting that this is a short‐term modification as longer HFHS diets do not lower fecal BA loss in rats and/or mice in our hands.

Interruption of entero‐hepatic recycling via BA sequestrants or inhibition of apical sodium‐dependent BA transporters (ASBT) increases fecal excretion of Chol and BA (Watanabe et al., 2012) and results in compensatory upregulation of hepatic ACLY, HMGCoAR, and CYP7a1 expression (Potthoff et al., 2013; Watanabe et al., 2012). BA sequestrants have been shown to lower lipids and glucose in humans (Beysen et al., 2012; Chiang, 2013; Handelsman, 2011) while both BA sequestrants and ASBT inhibition prevent or treat HFD‐induced hepatic steatosis in rodents (Wang et al., 2017; Watanabe et al., 2006; Watanabe et al., 2012). Considering our previous findings demonstrating HCR rats are protected against HFHS‐induced hepatic steatosis and our current study demonstrating HCR rats have greater Cyp7a1 expression in response to HFHS, we hypothesized HCR rats would exhibit greater fecal BA in response to acute HFHS. In contrast to our hypothesis, while HCR rodents displayed greater overall fecal BA content, both HCR and LCR animals exhibited similar reductions of fecal BA in response to a 3‐day acute HFHS. However, HCR rats had a lower total BA in hepatic portal serum (i.e., recycled BA) compared with their LCR counterparts which were not increased by acute HFHS, despite nutrient excess, whereas LCR rats exhibited large increases in hepatic portal serum BA following acute HFHS, suggesting HCR rats had greater BA loss. Collectively, our findings suggest acute HFHS‐induced increases in Cyp7a1 expression observed in HCR rats may be due, in part, to the interruption of the enterohepatic recycling of BA. Another interesting finding is that hepatic negative c‐repressor small heterodimer partner (SHP1) expression is increased at the same time that hepatic CYP7a1 is upregulated. BAs that are returned to the liver activate the nuclear receptor FXR which induces an increased expression of SHP1 which in turn downregulates CYP7a1 expression. This feedback inhibition mechanism is designed to limit excess BA production. Data showing that SHP1 and CYP7a1 are both elevated in HCR is unexpected and suggestive that SHP1 is less able to reduce CYP71a in HCR livers. This response could be tied to significant reductions in hepatic and systemic BA that is witnessed in the HCR, and adaptive response to maintain hepatic BA levels via increased CYP7a1 expression. It is also possible that a 3‐day HFHS has induced changes in BA metabolism that are in the midst of a highly adaptive process (see rapid changes in circulating and fecal BA levels) and thus a longer HFHS diet is needed to see homeostatic changes in FXR/SHP1 and CYP7a1 that track with the well‐known interactions of these regulatory pathways.

It should also be noted that high‐fat diets significantly reduce DNL flux measured by D_2_O tracer approaches, a hepatic response to the higher exogenous intake of fatty acids. As noted, the HFHS also lowered circulating BA and liver BA levels. This possibly suggests that the total flux of acetyl‐CoA toward both DNL and cholesterol/BA synthesis is blunted in HFHS conditions. Additional studies using tracer approaches to quantify BA synthesis rates in HCR/LCR rats along with DNL rates are needed to truly test these links. In addition, the human relevance of these papers is a critical component. We recently recruited women who were highly fit (due to regular endurance exercise) or moderately fit but matched for age and body mass to test if they displayed differences in BA metabolism and fecal loss (Maurer et al., 1985). We did not see direct evidence that BA synthesis was higher in the high‐fit women (measured by the BA synthesis marker, 7‐alpha‐hydroxy‐cholesten‐3‐one [C4]), however, we did see pronounced differences in BA metabolism in the high‐fit women during an oral glucose tolerance test which tracked with greater insulin sensitivity. We may have not seen increased BA synthesis in the high‐fit group because they were fed a eucaloric diet instead of hypercaloric. Moreover, we did not see evidence of differences in fecal BA loss, but we only obtained one stool sample and measured fecal BA concentration instead of collecting stool over a longer period and quantifying overall fecal BA loss. Thus, future research on the impact of aerobic capacity and exercise on BA metabolism in human subjects is needed.

Aerobic capacity can also be increased by chronic exercise which has also been shown to improve hepatic steatosis independent of weight status. However, the impact of exercise training on BA metabolism is limited. Previous studies have demonstrated VWR in mice is sufficient to increase fecal BA and cholesterol content (Meissner et al., 2010, 2011). Additionally, we have demonstrated that a 14‐week exercise and weight loss intervention in obese women improved C4, a marker of Cyp7a1 enzyme activity, and reduced systemic BA concentrations (Mercer et al., 2021). Our current study further supports this by demonstrating that 4 weeks of VWR results in greater fecal BA content in female C57BL6J mice and adds to the literature by demonstrating concomitant upregulation of genes involved in cholesterol and BA synthesis (i.e., HMGCR and Cyp7a1) in both female mice and male Sprague Dawley rats. While the exercise‐specific regulation of Cyp7a1 remains unknown, a recent study demonstrated genetic overexpression of transcription factor EB (TFEB) also robustly increases Cyp7a1 gene expression (Wang et al., 2020). Importantly, exercise has been shown to activate TFEB via nuclear translocation in skeletal muscle (Erlich et al., 2018), yet it remains unknown if this similarly occurs in the liver. Future studies should investigate the potential role of TFEB in the exercise‐specific regulation of hepatic Cyp7a1 and subsequent fecal BA excretion. Our overall findings demonstrate that extrinsic factors such as chronic exercise, a common therapeutic intervention for improving aerobic capacity, also increase fecal BA content and genes regulating BA synthesis.

Epigenetic mechanisms underlie transcriptional changes and fine‐tune gene expression (Blewitt & Whitelaw, 2013). A number of epigenetic processes are involved in the maintenance of transcriptional pathways including DNA methylation, histone modifications, and regulation via small and long non‐coding RNAs (Sharma & Rando, 2017). Here we examined differences in genomewide localization of key histone modifications between HCR and LCR groups. The choice of the specific modifications assessed was designed to encompass diverse gene regulatory functions (activation, repression, etc.) and genomic locations (promoter, enhancer, and heterochromatin). While this approach was a top‐down hypothesis generating, we focused these analyses in the context of gene expression changes observed between these groups. One salient finding from the analysis was that HCR rats showed enrichment of acetylation of histone (generally correlated with increased expression) and depletion of repressive histone marks among promoters (Grunstein, 1997). This pattern suggests alterations in underlying pathways regulating these marks (such as histone acetyltransferases and deacetylates, HATs, and HDACs). While this was not directly assessed in the present report, the hypothesis represents an important further direction to be explored which may explain differences in transcriptional responses between HCR and LCR rats. A second important feature of this report was the use of both histone mark enrichment and de novo genome segmentation methods (ChromHMM) that inform about complex chromatin states. Both approaches consistently suggest that genes that were upregulated in HCR rats following acute exposure to HFHS diets were also associated with robust gain in histone acetylation and a concurrent chromatin state transition to more active transcriptional states. Nevertheless, a deeper analysis of chromatin states and histone modifications following both acute and chronic HFHS in these diverging groups is warranted in the future.

In our current study, we demonstrate intrinsic aerobic capacity‐specific differing responses to acute nutrient excess despite all rats being completely sedentary. While our study provides important insight into initial adaptations, it is important to note that our 3‐day diet intervention is very brief and the impact of chronic nutrient excess on aerobic capacity‐specific adaptive responses to HFHS is necessary. Additionally, while our measures of total BA content in various tissues display differences based on aerobic capacity and HCR rats exhibited greater Cyp7a1, the rate‐limiting enzyme for BA synthesis, our current analysis does not provide any insight into synthesis rates for specific BA species. Importantly, the composition of BA is critical as it impacts the efficiency of re‐uptake and recycling which may be relevant considering different levels of fecal BA content observed in our current study.

In summary, we demonstrate animals with greater intrinsic aerobic capacity exhibit greater transcriptional adaptability to an acute HFHS diet intervention that may be partially driven by epigenetic differences in histone acetylation patterns. Microarray analysis, qPCR analyses, and metabolic measures point to a potential role of BA metabolism in the protection against acute nutrient excess exhibited by HCR rats. We interpret our collective findings to suggest BA excretion and subsequent increases in BA synthesis may serve as an alternate pathway to de novo lipogenesis for excess nutrients and act as an energetic siphon to protect HCR rats from acute HFHS‐induced hepatic steatosis when compared with their LCR counterparts. Future studies are needed to directly quantify metabolic flux through DNL and BA metabolism in addition to studies that mechanistically determine the role of enhanced BA in the metabolic protection associated with exercise.

## AUTHOR CONTRIBUTIONS

Harrison D. Stierwalt, R. Scott Rector, and John P. Thyfault conception and design of research; Lauren G. Koch and Steven L. Britton provided the underlying Energy Transfer Hypothesis and the LCR and HCR rats; E. Matthew Morris and Grace M.E. Meers performed experiments; Harrison D. Stierwalt, E. Matthew Morris, Greg Graf, Kelly Mercer, Kartik Shankar, and John P. Thyfault analyzed data; Harrison D. Stierwalt and John P. Thyfault interpreted results; Harrison D. Stierwalt and Kartik Shankar prepared figures; Harrison D. Stierwalt drafted the manuscript; Harrison D. Stierwalt, E. Matthew Morris, Adrianna Maurer, Udayan Apte, Tiangang Li, Greg Graf, R. Scott Rector, Kelly Mercer, Kartik Shankar, and John P. Thyfault edited and revised the manuscript; Harrison D. Stierwalt and John P. Thyfault approved the final version of the manuscript.

## FUNDING INFORMATION

This work was supported by the National Institutes of Health (NIH) (grant nos. DK088940‐01A1 [J.P.T.], R01DK121497 [J.P.T.], 5T32AR48523‐8 [E.M.M.], and F32DK130244 [H.D.S.]), the American Heart Association (AHA) (grant no. 14POST20110034 [E.M.M.]), and a VA Merit Grant. The HCR/LCR rat models were funded by the Office of Research Infrastructure Programs/OD Grant P40OD021331 from the NIH (L.G. Koch and S.L. Britton). H.D. Stierwalt is supported by F32DK130244 from the NIH, R.S. Rector is supported by VA Merit (grant no. I01BX003271), and J.P. Thyfault is supported by VA Merit (grant no. 1I01BX002567‐05) along with NIH grants R01DK121497, R01AR071263, and 1R01AG069781. This work was supported by resources and the use of facilities at the Harry S. Truman Memorial VA Hospital in Columbia, MO. Contact L.G.K. (Lauren.Koch2@UToledo.Edu) or S.L.B. (brittons@umich.edu) for information on the LCR and HCR rats: these rat models are maintained as a research resource at the University of Toledo, Toledo, Ohio.

## CONFLICTS OF INTEREST

The authors have no conflicts of interest, financial, or otherwise to disclose for this research.

## ETHICAL STATEMENT

Institutional and national guidelines for the care and use of animals were followed.

## Supporting information


Figure S1

Table S1
