# Supplementary material for: Rats with high aerobic capacity display enhanced transcriptional adaptability and upregulation of bile acid metabolism in response to an acute high‐fat diet
Source: Physiol Rep. 2022 Aug 3;10(15):e15405. doi: 10.14814/phy2.15405 (PMC9350427; doi:10.14814/phy2.15405)

**Supplementary Figure 1.** HFD induces rapid reductions in tricaboxylic acid (TCA) cycle genes in LCR rats but not HCR rats. Gene expression heat map data are presented for TCA Cycle genes from the analysis of pooled (n = 3) liver mRNA from LCR and HCR rats fed the 45% HFD for 3 days. Red represents greater expression and blue represents lower expression.


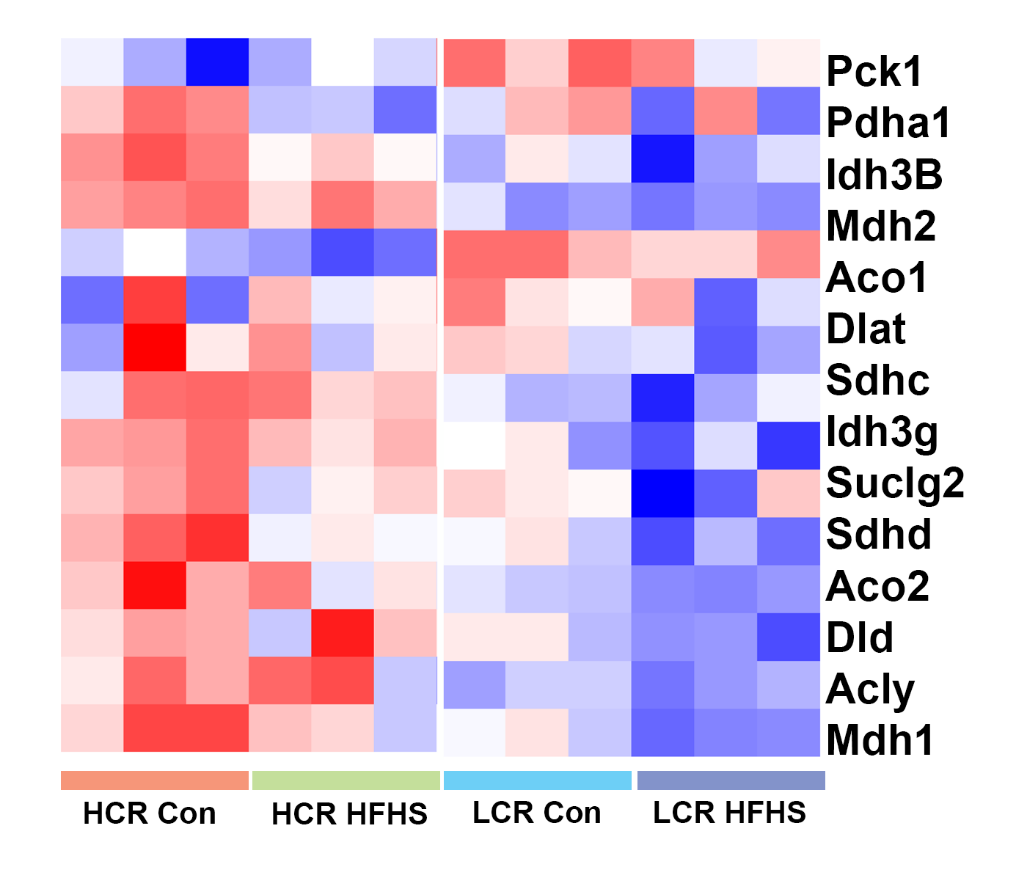

Supplement: Supplementary file 1 — Figure S1 [file PHY2-10-e15405-s002.docx]
