# Supplementary material for: Rats with high aerobic capacity display enhanced transcriptional adaptability and upregulation of bile acid metabolism in response to an acute high‐fat diet
Source: Physiol Rep. 2022 Aug 3;10(15):e15405. doi: 10.14814/phy2.15405 (PMC9350427; doi:10.14814/phy2.15405)
Supplement: Supplementary file 2 — Table S1 [file PHY2-10-e15405-s001.docx]

| Supplementary Table 1. SYBR RT-PCR primers | | |
| --- | --- | --- |
| Gene | Forward Primer | Reverse Primer |
| GK | TCTACTTCCCCAACGACCCCT | GTTCATGCCCGTTGTGAGT |
| ACLY | GCTTACGGACAGAGAGCCAC | GTCTGTCGGGAGTAACCC |
| HMGCR | GCCTCCATTGAGATCCGGAGG | AGGGATGGGAGGCCACAAAG |
| Cyp7a1 | GCGAAGGCATTTGGACACAG | ACCCAGGCATTGCTCTTTGA |
| SQLE | GATGCCGCTATTTTCCAGGC | CTTTTCGGAGCTGACGCAAG |
| BSEP | TGGGGCTCGTCAGATAAGGA | ACATGCGCTGGAGGAAATGA |
| FXR | CATTACAACGCGCTCACCTG | TTCCTTAGCCGGCAATCCTG |
| SHP | CTGCCTGGAGTCTTTCTGGA | TCCAGGACTTCACACAATGC |
| CycB (PPIB) | CTTAGCTACAGGAGAGAAAGG | TTCAGCTTGAAGTTCTCCATC |
